# Dynamic Prediction of Overall Survival for Patients with Osteosarcoma: A Retrospective Analysis of the EURAMOS-1 Clinical Trial Data

**DOI:** 10.3390/curroncol31070267

**Published:** 2024-06-22

**Authors:** Marta Spreafico, Audinga-Dea Hazewinkel, Hans Gelderblom, Marta Fiocco

**Affiliations:** 1 Mathematical Institute, Leiden University, Einsteinwg 55, 2333 CC Leiden, The Netherlands; m.fiocco@math.leidenuniv.nl; 2 Department of Biomedical Data Sciences—Medical Statistics, Leiden University Medical Center, Albinusdreef 2, 2333 ZA Leiden, The Netherlands; 3 Department of Medical Statistics, Faculty of Epidemiology and Population Health, London School of Hygiene & Tropical Medicine, Keppel Street, London WC1E 7HT, UK; dea.hazewinkel@lshtm.ac.uk; 4 Department of Medical Oncology, Leiden University Medical Center, Albinusdreef 2, 2333 ZA Leiden, The Netherlands; a.j.gelderblom@lumc.nl; 5 Trial and Data Center, Princess Maxima Center for Pediatric Oncology, Heidelberglaan 25, 3584 CS Utrecht, The Netherlands

**Keywords:** dynamic prediction, osteosarcoma, clinical trial, landmark analysis, survival

## Abstract

Current prediction models for patients with ostosarcoma are restricted to predictions from a single, static point in time, such as diagnosis or surgery. These approaches discard information which becomes available during follow-up and may have an impact on patient’s prognosis. This study aims at developing a dynamic prediction model providing 5-year overall survival (OS) predictions from different time points during follow-up. The developed model considers relevant baseline prognostic factors, accounting for where appropriate time-varying effects and time-varying intermediate events such as local recurrence (LR) and new metastatic disease (NM). A landmarking approach is applied to 1965 patients with high-grade resectable osteosarcoma from the EURAMOS-1 trial (NCT00143030). Results show that LR and NM negatively affected 5-year OS (HRs: 2.634, 95% CI 1.845–3.761; 8.558, 95% CI 7.367–9.942, respectively). Baseline factors with strong prognostic value (HRs > 2) included poor histological response (≥10% viable tumor), axial tumor location, and the presence of lung metastases. The effect of poor versus good histological response changed over time, becoming non-significant from 3.25 years post-surgery onwards. This time-varying effect, as well as the strong impact of disease-related time-varying variables, show the importance of including updated information collected during follow-up in the model to provide more accurate survival predictions.

## 1. Introduction

Osteosarcoma is the most common primary bone cancer and mainly affects children, adolescents, and young adults [1,2,3]. Since the mid-1980s, little progress has been made in improving the survival of patients diagnosed with osteosarcoma [1,4,5]. Current management strategies combine neoadjuvant multi-agent chemotherapy, surgical removal of the primary tumor and, if resectable, all metastatic disease, followed by adjuvant chemotherapy [5].

In 2001, the EURAMOS (European and American Osteosarcoma Studies) collaboration was established, with the purpose of pooling resources and facilitating the study of osteosarcoma [6]. Recruitment for the first trial—the EURAMOS-1 clinical trial (NCT00143030)—took place from 2005 to 2011 yielding a total of 2260 patients, a subset of which (1334) were randomized to treatment [7]. The effect of various prognostic factors on event-free and overall survival has previously been examined for this trial [3,8,9]. Analyses, however, have been restricted to the randomized patient cohorts [8,9] and/or limited to considering factors set at diagnosis or at baseline [3,8,9]. A recent re-analysis based on multistate models quantified the impact of experiencing specific disease stages (e.g., the development of local recurrence or metastatic disease), conditional on prior events and patient characteristics [10]. Multistate model estimation, however, is in practice limited to transitions with a sufficient number of events per predictor category.

In this study, an alternative analysis of the EURAMOS-1 data is proposed using a dynamic prediction model developed trough a landmarking approach [11,12]. Three main advantages of a dynamic prediction model can be distinguished. First, a notable drawback of classic survival models is that predictions are made from baseline—i.e., the start of follow-up—and that all covariates are also considered at baseline and assumed to have a constant effect on patient survival. Dynamic prediction models consider various prediction time points, $t_{p}$, making it possible to obtain predictions for patients who have survived for a given time since the start of follow-up. For example, a patient who has already survived for three years may be expected to have a different prognosis than a patient who has been observed to survive for only one year. Second, a time effect can also be ascribed to covariates measured at baseline. For instance, a covariate may have a stronger effect at the beginning of follow-up. Dynamic prediction models offer a third and final advantage in the potential for the inclusion of time-varying covariates, allowing one to model the effect of various events occurring after the start of follow-up. This makes it possible to update a patient’s prognosis given the occurrence of an intermediate event, such as a local recurrence or new metastatic disease. Such intermediate events are relevant in survival prediction yet cannot be included in a straightforward Cox regression [13]. The relevance of a dynamic prediction model has previously been demonstrated in other oncological studies, such as for breast cancer [14] and high-grade soft tissue sarcoma [15,16].

The primary aim of this study was to develop a dynamic prediction model capable of providing 5-year overall survival (OS) predictions from various specific time points $t_{p}$ (up until 5 years after surgery). The landmark model, built using the full EURAMOS-1 cohort of patients who underwent surgery, incorporates baseline factors along with evolving disease-related variables and accounts for time-varying covariate effects. Patients characterized by specific characteristics are employed to illustrate the outcomes of the prediction model.

## 2. Materials and Methods

### 2.1. Study Design

The EURAMOS collaboration was established in 2001 between the Children’s Oncology Group (COG), the Cooperative Osteosarcoma Study group (COSS), the European Osteosarcoma Intergroup (EOI), and the Scandinavian Sarcoma Group (SSG) [6]. From 2005 to 2011, a total of 2260 patients aged 40 or younger with a newly diagnosed resectable osteosarcoma were recruited in the EURAMOS-1 clinical trial (NCT00134030) [7]. Patients underwent neadjuvant chemotherapy prior to resection of the primary tumor. Histological response was assessed in the resected specimen and classified as poor (≥10% viable tumor) or good (<10% viable tumor). In total, 1334 (59%) patients were randomized for treatment: patients with a poor histological response received MAP (methotrexate, doxorubicin, cisplatin) or MAPinf (MAP plus ifosfamide and etoposide), while patients with a good histological response were allocated MAP or MAPIE (MAP by pegylated interferon). An extensive description of the trial and treatment protocol has been provided previously [7]. The primary analysis found no beneficial effect of experimental treatment in either group [8,9].

### 2.2. Patients and Variables

**Figure 1:**
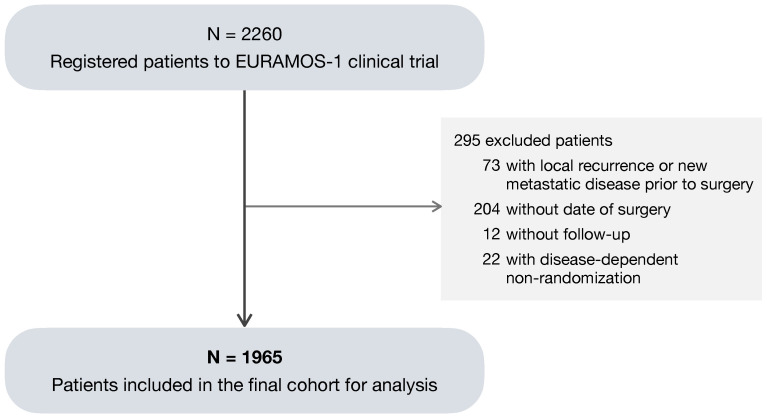
Consort diagram of patients included in the analysis.

Of the total 2260 patients registered to EURAMOS-1, a subset of 1965 patients (87%) was considered eligible for the analysis (Figure 1). The 295 (13%) excluded patients met one or more of the following criteria: (1) a local recurrence or new metastatic disease recorded prior to the date of primary surgery; absence of (2) a surgery date or (3) follow-up; and (4) non-randomization due to progression of metastatic disease, the development of a new metastatic disease, or the presence of an unresectable disease. As no benefit of experimental treatment was found in the primary analysis [8,9], both randomized and non-randomized patients were included in the analysis in order to make the best use of the available data.

The EURAMOS-1 trial data include predictor measurements and records of intermediate events observed during follow-up. Ten predictors of interest were selected for the analysis. Predictors measured at baseline included age at primary surgery (years), sex (male, female), tumor location (proximal femur/humerus, axial, other), absolute tumor volume (cm^3^), surgical excision as reported by the pathologist (wide/radical, marginal, intralesional/unknown), the presence of lung metastases (no, yes/possible), the presence of other metastases (no, yes/possible), and histological response as assessed in the resected specimen of the primary tumor (good: <10% viable tumor; poor: ≥10% viable tumor). Three age groups were defined according to the classification introduced by Collins et al. [17]: child (male: 0–12 years; female: 0–11 years), adolescent (male: 13–17 years; female: 12–16 years), and adult (male: 18 or older; female: age 17 years or older). Tumor location (proximal femur/humerus, axial, other) was defined according to the definition used in previous analyses of survival and prognosis in the EURAMOS-1 trial [3] by pooling study variables “site” (femur, tibia, fibula, humerus, radius, ulna, scapula/clavicle, pelvis/sacrum, rib, spine, other) and “location” (proximal, diaphysis, distal, N/A not long bone). A good histological response and the presence of metastases have previously been found to be associated with improved and decreased survival, respectively [4,18,19,20,21,22]. In addition to baseline covariates, the disease-related intermediate events of (i) local recurrence (LR) of the primary tumor and (ii) occurrence of a new metastatic (NM) disease were included in the model developed in this study as time-varying binary covariates (no, yes). By combining these two time-varying covariates for LR and NM, four different time-varying patient disease statuses can be obtained: a patient experienced (1) no intermediate event, (2) only local recurrence, (3) only new metastatic disease, or (4) both.

### 2.3. Statistical Analysis

Overall survival (OS) was measured in years since date of surgery. The reverse Kaplan–Meier method [23] was used to estimate median OS. The effects of risk factors on dynamic OS were evaluated by employing a proportional landmark supermodel [11,12] to dynamically predict the 5-year probability of death at different prediction time points during follow-up. Dynamic prediction can be defined as the making of a prediction at a specific time, named landmark time point $t_{LM}$, given the entire history of events and covariates up to that time. This is carried out by selecting all the individuals at risk at that time and using solely the information available at that specific time point to make the prediction. For each landmark time point, $t_{LM}$, a Cox proportional hazards model [13] is estimated using all patients alive and in follow-up at time, $t_{LM}$. All these individual Cox models are subsequently combined into a single landmark supermodel, which can make predictions at a range of time points $t_{p}$ for a set prediction window [11,12]. For this analysis, landmark time points were chosen in increments of 3 months from 0 to 5 years after surgery. For the time-varying covariates LR and NM, the patient status was updated at each landmark time. The remaining prognostic factors, measured at time of surgery (baseline), were kept constant. A prediction window *w* of 5 years was defined with the purpose of obtaining the 5-year death probabilities as predicted at different times $t_{p}$ in the first 5 years of follow-up.

Linear and quadratic time effects were included in the model, denoted $t_{LM}$ and $t_{LM}^{2}$, respectively. As histological response may have a time-varying effect on survival [10], its main time-constant effect was modeled along with the associated linear and quadratic time-varying effect. In order to incorporate the latter two in the model, interactions between histological response covariate and $t_{LM}$ and $t_{LM}^{2}$ were included.

To assess the validity of the prognostic model, a heuristic shrinkage factor was calculated as a measure of internal calibration [12]. A well-calibrated model ensures small differences between observed and predicted survival probabilities. The heuristic shrinkage factor, used in the case that no external data set is available for validation, gives a measure for calibration by indicating to what extent the regression coefficients should be shrunk towards the mean to ensure the model generalizes well to new data. Its value ranges from 0 to 1, where a value approaching 1 suggests a good calibration.

The discriminative ability of the model was measured using a cross-validated dynamic concordance index (C-index) [12,24]. A model with a good discriminative ability predicts higher risk for patients that experience an event earlier. The C-index is defined as the proportion of pairs for which the order of observed survival times is matched by the order of model predictions. A C-index of 0.5 indicates no discriminative ability, while a C-index of 1 indicates perfect discrimination. In the dynamic setting, with a prediction window of w=5 years, the C-index at time *t* was obtained by considering event times within the window [t;t+w]. The dynamic C-index was computed at different times t=0,1,2,3,4,5 (in years) after surgery, using a leave-one-out cross-validation approach [25].

Of the 1965 patients included in the analysis, 394 (20%) had missing values for one or more covariates. The highest percentage of missing (17.7%) was observed for the prognostic factor absolute tumor volume. To make optimal use of the available data, missing values for tumor volume and histological response were imputed in a 10-fold multiple imputation approach, using the R-package Amelia II [26]. The analysis was applied to each of the 10 complete datasets, and results were pooled using Rubin’s rule [27]. All analyses were performed in the R-software environment (R version 4.3.1) [28].

## 3. Results

**Figure 2:**
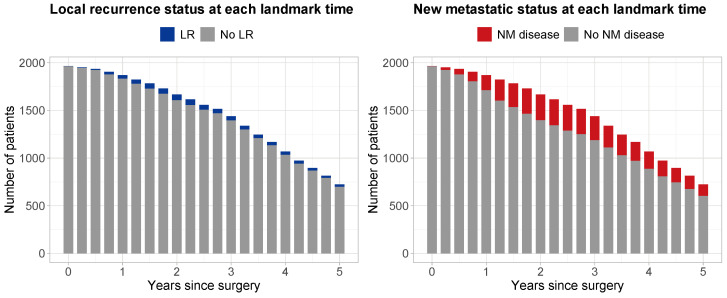
Number of patients at risk at each landmark time point $t_{LM}$ from 0 to 5 years after surgery with increments of 3 months. Left panel: blue, patients with local recurrence; gray, patients with no local recurrence. Right panel: red, patients with a new metastatic disease; gray, patients without a new metastatic disease.

A total of 1965 patients were considered to be eligible for analysis. Median follow-up time since surgery, estimated with reverse Kaplan–Meier [23], was 4.96 years (95% CI 4.87–5.08). At the end of study, 466 (23.7%) patients had died, while 1499 (76.3%) remained alive. Table 1 shows the characteristics at surgery for all patients, the 1328 randomized patients, and the 637 non-randomized patients. Figure 2 shows the number of patients considered at risk at each landmark time $t_{LM}$, along with their LR status (left panel—gray: no LR; blue: with LR) and their NM disease status (right panel—gray: no NM; red: with NM). During follow-up, a total of 130 (6.6%) patients experienced LR, and 570 (29%) patients NM.

### 3.1. Dynamic Prediction Model

The effect of the eight baseline predictors (Table 1) and the two time-varying covariates for LR and NM (Figure 2) on OS was assessed using proportional landmark supermodels. The hazard ratios (HRs) and 95% confidence intervals (95% CIs) estimated from the dynamic model are shown in Table 2. All predictors had significant time-constant effects. The strongest prognostic factors were the occurrence of NM disease, with a HR of 8.558 (95% CI: 7.367–9.942), followed by LR, with a HR of 2.634 (95% CI: 1.845–3.761). The presence of lung metastases and other metastases gave HRs of 2.177 (95% CI: 1.832–2.587) and 1.860 (95% CI 1.383–2.501), respectively. A poor versus good histological response was associated with a decrease in OS (HR: 2.371; 95% CI: 2.020-2.783). An axial tumor site, compared to any other limb site except proximal femur/humerus, resulted in a HR of 2.071 (95% CI 1.475–1.492). An intralesional/unknown excision, compared to a wide/radical one provided a HR of 1.423 (95% CI: 1.041–2.945). The remaining predictor categories had more modest effects with hazard ratios smaller than 1.25. Finally, being a child was found to be associated with increased OS compared to being an adolescent, resulting in a good prognostic factor with an HR of 0.712 (95% CI: 0.575–0.881).

Notably, histological response had a significant time-varying effect. This effect was modelled by an additional linear time component and quadratic time component with HRs of 0.728 (95% CI: 0.641–0.826) and 1.036 (95% CI: 1.001–1.072), respectively. The hazard ratio for a patient with poor versus good histological response was calculated using the following formula:

(1) $$HR\left(t_{p}\right)=HR_{CON}\times HR_{LIN}^{t_{p}}\times HR_{QUAD}^{t_{p}^{2}}=2.371\times 0.728^{t_{p}}\times 1.036^{t_{p}^{2}},$$

where $HR_{CON}$ denotes the time-constant component, $HR_{LIN}$ the linear time-varying component, $HR_{QUAD}$ the quadratic time-varying component, and $t_{p}$ the prediction time in years since surgery.

**Figure 3:**
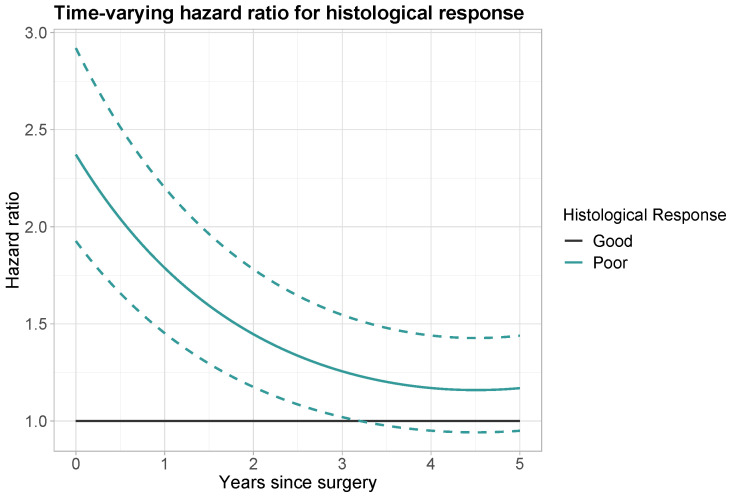
Time-varying hazard ratio for histological response (black: good histological response; aquamarine: poor histological response). Dashed lines: point-wise 95% confidence interval for poor histological response.

The calculations for prediction times $t_{p}$ of 1, 2, 3, 4 and 5 years are reported in Table 3. Directly after surgery the hazard ratio for poor histological response was equal to the time-constant effect of 2.371. At prediction time $t_{p}=1$ year, the HR has decreased to 1.788. At prediction time $t_{p}=3$ years, the HR has further decreased to 1.256. A visual representation of the time-varying hazard ratio is given in Figure 3. The decrease in HR over time is at first more steep, then more gradual. From 3.25 years onwards, a HR of 1 falls within the 95% confidence interval and a poor histological response (versus good histological response) is no longer a significant predictor for OS.

### 3.2. Model Calibration Furthermore, Discrimination

The estimated heuristic shrinkage factor of 0.997 indicated that the model is very well calibrated. To assess the discriminative ability of the model, dynamic leave-one-out cross-validated C-indeces were computed at six time points *t* using a prediction window of w=5 years. C-index values were equal to 0.693, 0.777, 0.837, 0.837, 0.830, and 0.847 at times t=0,1,2,3,4,5 years since surgery. These high values indicated a very good discriminative ability of the model.

### 3.3. Dynamic Predictions of 5-Year Death Probability for Selected Patients

**Figure 4:**
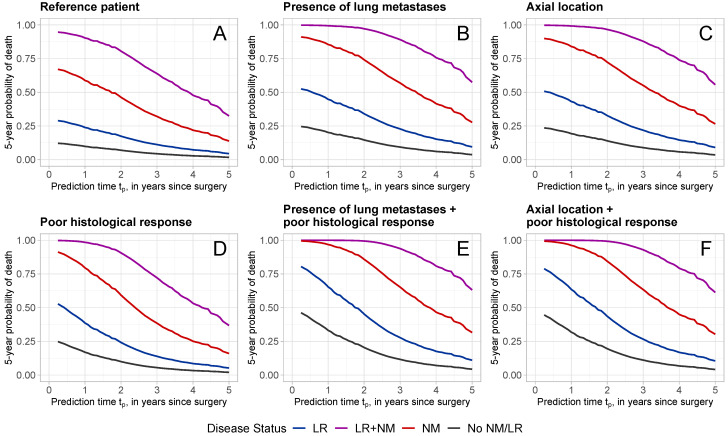
Five-year death probabilities for patients with different characteristics and disease status. Patient characteristics are reported in Table 4. Different colors refer to different disease statuses (black: No LR/NM; blue: with LR; red: with NM; purple: with LR + NM).

Figure 4 displays the probabilities of dying within 5 years at different time points $t_{p}$ (from 3 months to 5 years after surgery) for patients with specific characteristics and different disease progression status. The specific characteristics for each patient are provided in Table 4. Patient A is considered the “*reference*” and refers to a patient with reference values for the predictors evaluated at surgery: an adolescent female patient with a good histological response, no lung metastases or other metastases, a wide/radical surgical excision, and a tumor with absolute volume ≤ 200 cm^3^, located at any site except axial skeleton and proximal femur/humerus. The remaining patients B–F were defined in terms of characteristics that diverge from those of the reference patient A. For each patient profile A–F, predictions were made given that the patient experienced no intermediate event (black line: no NM/LR), experienced a local recurrence (blue line: with LR), a new metastatic disease (red line: with NM), or both (purple line: with LR + NM). For all patients, the occurrence of NM and/or LR increased the 5-year probability of dying drastically.

Figure 4A shows the 5-year death probabilities for reference patient A. Given that the patient is alive at $t_{p}=1$ year after surgery, the probability of dying within 5-years, given that no LR or NM has occurred is 9.8%. This probability increased with the occurrence of LR, NM, or LR + NM to 23.9%, 58.8%, and 90.3%, respectively. Poor histological response (Figure 4D) increased the probability of dying noticeably, with a predicted probability of 16.9% at 1 year for no LR/NM, 38.6% for LR, 79.5% for NM, and 98.5% for LR + NM. The presence of lung metastases (Figure 4B) or axial tumor location (Figure 4C) also increased the 5-year death probabilities. The combination of poor histological response with the presence of lung metastases (Figure 4E) or an axial tumor location led to even higher 5-year death probabilities.

For all patients, the later the prediction time, the lower the 5-year probability of dying. Consider, for example, patient D with a poor histological response, who experienced NM (red line in Figure 4D) at prediction times of $t_{p}=1,3,5$ years. At 1 year, the probability of dying within 5 years was 79.5%, which has decreased to 28.5% and 16.0% at 3 and 5 years, respectively.

## 4. Discussion

The dynamic prediction model developed in this study can be used to obtain predictions of the 5-year survival probability from different time-points, starting from the time of surgery, up to 5 years after surgery. The model considers a range of relevant covariates, accounting, where appropriate, for time-varying effects, and including various major disease-related events that may occur during follow-up.

Our study shows that the dynamic prediction model captures relevant information that would not be available with a static approach. For instance, the prediction time, in years from surgery, strongly influences the probability of dying within 5 years, with the 5-year death probability decreasing the longer a patient survives. Patient information was updated over time through the dynamic variables LR and NM disease. Both were found to be associated with a high increase in the 5-year death probabilities, with hazard ratios of 2.634 and 8.558, respectively. Confirming previous reports [3,4,10,18,19,20,21,22], a poor histological response, an axial tumor location, and the presence of lung metastases were strong risk predictors for poor OS. Of interest is that undergoing an intralesional/unknown surgical excision was found to be associated with a decreased OS compared to a wide/radical excision (HR = 1.423). Unlike the other predictors considered, excision is not an observational variable and could be subject to intervention.

For the histological response, a significant time-varying effect was found. The hazard ratio for a poor histological response versus a good one decreases from 2.371, at the time of surgery, to 1.169, at a prediction time of 5 years, with no significant effect observed from 3.25 years onwards. This indicates that directly after surgery histological response is a strong predictor, but it becomes weaker with time. This finding suggests that it would be relevant to examine potential time effects of histological response in other datasets, as it is commonly considered an important predictor in the clinical framework.

The quality of the prediction model was assessed with regards to time conform the endorsement criteria for risk models, as defined by the American Joint Committee on Cancer [29]. Internal validation was performed by means of cross-validation, a heuristic shrinkage factor was employed to confirm good internal calibration, and dynamic cross-validated C-indices were used to establish good discriminative ability over time.

This analysis has some limitations. Some covariates have unbalanced sizes among the different categories (e.g., tumor location), and the occurrence of LR is observed only in 130/1965 (6.6%) patients. Therefore, some caution is required when interpreting the results. By necessity, a simplified model is presented, with disease progression prior to death characterized by the first occurrence of intermediate events LR and/or NM, and repeated occurrences of NM (N = 71) and LR (N = 1) discarded.

## 5. Conclusions

The dynamic prediction model developed in this study, using almost 2000 patients from the EURAMOS-1 clinical trial, can be used to make reliable predictions of the probability of dying within 5 years from a given prediction time, which can be any time from surgery up to 5 years after surgery. This dynamic model is a highly relevant addition to existing models, as it accounts for patient status changing over time and, unlike a static model, gives updated predictions for a range of times. To the best of the authors’ knowledge, this is the first study where dynamic OS for patients with osteosarcoma is investigated. Reanalyzing data from large randomized studies, using the dynamic landmarking approach, may also yield valuable insights for patients with other oncological conditions, as shown also for breast cancer [14] or high-grade soft tissue sarcoma [15,16].

## Figures and Tables

**Table 1:** Patient demographics, tumor, and treatment characteristics at surgery.

	Treatment Randomization					
	Randomized		Not Randomized		Total	
Predictor	N	%	N	%	N	%
Age ^1^						
Adolescent	670	50.5	309	48.5	979	49.8
Child	286	21.5	155	24.3	441	22.4
Adult	372	28.0	173	27.2	545	27.8
Sex						
Female	546	41.1	264	41.4	810	41.2
Male	782	58.9	373	58.6	1155	58.8
Tumor location ^2^						
Other	1131	85.2	510	80.1	1641	83.5
Axial	37	2.8	27	4.2	64	3.3
Proximal femur/humerus	160	12.0	100	15.7	260	13.2
Volume ^3^						
<200	734	55.3	363	57.0	1097	55.8
≥200	341	25.7	180	28.3	521	26.5
Missing	253	19.0	94	14.8	347	17.7
Excision						
Wide/Radical	1096	82.5	510	80.1	1606	81.7
Marginal	175	13.2	63	9.9	238	12.1
Intralesional/Unknown	57	4.3	64	10.0	121	6.2
Lung metastases						
No	1083	81.6	504	79.1	1587	80.8
Yes/Possible	245	18.4	133	20.9	378	19.2
Other metastases						
No	1279	96.3	610	95.8	1889	96.1
Yes/Possible	49	3.7	27	4.2	76	3.9
Histological response ^4^						
Good	715	53.8	281	44.1	996	50.7
Poor	609	45.9	306	48.1	915	46.6
Missing	4	0.3	50	7.8	54	2.7
Total	1328		637		1965	

^1^ Age groups were defined according to Collins et al. [17]: child (male: 0–12 years; female: 0–11 years), adolescent (male: 13–17 years; female: 12–16 years) and adult (male: 18 or older; female: age 17 years or older). ^2^ Tumor location (proximal femur/humerus, axial, other) was defined according to the definition used in previous analysis of survival and prognosis in the EURAMOS-1 trial [3,10]. Information was pooled from study variables “site” (femur, tibia, fibula, humerus, radius, ulna, scapula/clavicle, pelvis/sacrum, rib, spine, other) and “location” (proximal, diaphysis, distal, N/A not long bone). Observed axial tumor locations included rib (15) and pelvis/sacrum (49). ^3^ Absolute volume is measured in cm × cm × cm × 0.54. ^4^ A good and poor histological response is defined by the amount of tumor remaining after resection: <10% and ≥10% constitute a good and poor response, respectively.

**Table 2:** Dynamic prediction model: hazard ratios (HRs) and 95% confidence intervals (N = 1965).

	HR	95% CI	*p*-Value
Age			
Adolescent	1		
Child	0.744	0.605–0.914	0.005
Adult	0.949	0.788–1.142	0.578
Sex			
Female	1		
Male	1.205	1.023–1.420	0.025
Tumor location			
Other	1		
Axial	2.071	1.475–2.906	<0.001
Proximal femur/humerus	1.198	0.962–1.493	0.107
Absolute tumor volume			
<200 cm^3^	1		
≥200 cm^3^	1.255	1.057–1.489	0.009
Excision			
Wide/Radical	1		
Marginal	0.914	0.724–1.154	0.450
Intralesional/Unknown	1.423	1.041–1.945	0.027
Presence of lung metastases			
No	1		
Yes/Possible	2.177	1.832–2.587	<0.001
Presence of other metastases			
No	1		
Yes/Possible	1.860	1.383–2.501	<0.001
Histological Response			
Good	1		
Poor—Constant	2.371	2.020–2.783	<0.001
Poor—Linear time-varying effect	0.728	0.641–0.826	<0.001
Poor—Quadratic time-varying effect	1.036	1.001–1.072	0.042
Local Recurrence (LR)			
No	1		
Yes	2.634	1.845–3.761	<0.001
New metastatic disease (NM)			
No	1		
Yes	8.558	7.367–9.942	< 0.001
Follow-up time (ref: time of surgery)			
Linear *t*	0.675	0.620–0.735	<0.001
Quadratic $t^{2}$	1.047	1.028–1.066	<0.001

HR, hazard ratio; CI, confidence interval.

**Table 3:** Time-varying hazard ratio HR($t_{p}$) for 5-year dynamic OS for a patient with poor histological response at different prediction time points $t_{p}=1,2,3,4,5$.

$t_{p}$	Constant	Linear Time-Varying	Quadratic Time-Varying	HR($t_{p}$)	95% CI
1	2.371	$0.728^{1}$	$1.036^{1}$	1.788	1.452–2.201
2	2.371	$0.728^{2}$	$1.036^{4}$	1.446	1.175–1.781
3	2.371	$0.728^{3}$	$1.036^{9}$	1.256	1.020–1.546
4	2.371	$0.728^{4}$	$1.036^{16}$	1.170	0.950–1.440
5	2.371	$0.728^{5}$	$1.036^{25}$	1.169	0.949–1.439

HR, hazard ratio; CI, confidence interval; $t_{p}$, prediction time-point.

**Table 4:** Characteristics of patients A–F in Figure 4.

Patient	Age ^1^	Sex	Tumor	Excision	Volume ^1^	Lung	Other	Histological
			Location ^1^			Metastases	Metastases	Response ^1^
A (reference)	Adolescent	Female	Other	Radical/wide	<200	No	No	Good
B	Adolescent	Female	Other	Radical/wide	<200	Yes/Possible	No	Good
C	Adolescent	Female	Axial	Radical/wide	<200	No	No	Good
D	Adolescent	Female	Other	Radical/wide	<200	No	No	Poor
E	Adolescent	Female	Other	Radical/wide	<200	Yes/Possible	No	Poor
F	Adolescent	Female	Axial	Radical/wide	< 200	No	No	Poor

^1^ See Table 4 for detailed definition.

## Data Availability

Data may be obtained from a third party and are not publicly available. A request to access the EURAMOS-1 trial data may be submitted to the MRC Clinical Trials Unit (CTU, London). The application requires completion of an analysis and data release request form, where the applicant provides a project summary (detailing the motivation of the data request, the background and objectives of their project, and the reasons for requesting this specific dataset), the data requirements (for this study, the anonymized individual-level data for all registered patients were requested, including (demographic) patient characteristics, disease characteristics, pathology and surgical information, treatment data, and major events), and details on the proposed publication, authorship and acknowledgments policy. Data applications are submitted to the Coordinating Data Center (CDC, London), and subject to review by the Trial Management Group and the Trial Steering Committee.

## Abbreviations

The following abbreviations are used in this manuscript:

CI	Confidence interval
EURAMOS	European and American Osteosarcoma Studies
HR	Hazard ratio
LR	Local recurrence
NM	New metastatic disease
OS	Overall survival
